# Use of a Clinical Audit System in Implementing Surviving Sepsis Campaign Guidelines in Patients With Peritonitis

**DOI:** 10.7759/cureus.15961

**Published:** 2021-06-27

**Authors:** Ramya C Valiveru, Anusha Cherian, Krishnamachari Srinivasan, Nanda K Maroju

**Affiliations:** 1 Surgery, Jawaharlal Institute of Postgraduate Medical Education and Research, Puducherry, IND; 2 Anaesthesiology and Critical Care, Jawaharlal Institute of Postgraduate Medical Education and Research, Puducherry, IND

**Keywords:** surviving sepsis guidelines, audit, compliance, process improvement, peritonitis

## Abstract

Background

Sepsis is the predominant cause of morbidity and mortality in patients with peritonitis. "Surviving Sepsis Campaign" (SSC) is an international effort in reducing mortality based on evidence-based guidelines. This study aims to assess the impact of audit-based feedback in a Plan-Do-Study-Act (PDSA) format on improving the implementation of the SSC guidelines in patients with generalized peritonitis at our center.

Methods

This prospective observational study was conducted in four audit cycles in PDSA format. Multi-departmental inputs were taken to suggest modifications in practice. A questionnaire-based analysis of reasons for non-compliance was performed to find out the opinions and reasons for non-compliance. Morbidity, mortality, and the length of ICU and hospital stay among these patients were also analyzed.

Results

Baseline compliance with intravenous (IV) bolus administration, central venous pressure (CVP)-guided fluids, and inotropes support when indicated were 100%. Over the course of the three audit cycles, statistically significant improvement in compliance was noted for obtaining blood cultures before antibiotics, antibiotic administration within three hours of presentation, and serum lactate measurement. Overall bundle compliance improved from 9.2% to 64.7% by the end of audit cycle III.

Conclusions

This study demonstrates that audit-based feedback is a dependable means of improving compliance with SSC guidelines. It brings about improvement by educating users, modifying their behavior through feedback, and enhances process improvement by identifying and correcting systemic deficiencies in the organization.

## Introduction

Sepsis is the predominant cause of mortality and morbidity in patients with peritonitis [1-4]. "Surviving Sepsis Campaign" (SSC) guidelines are a set of evidence-based recommendations, tailored for bedside implementation that was proposed in 2004 to combat sepsis. These were popularized as time stipulated bundles for resuscitation and maintenance [5]. Their implementa­tion has proven to decrease mortality in severe sepsis and septic shock by many stud­ies worldwide [6-8]. However, various studies have identified poor compliance with these guidelines among practitioners [9].

Lack of awareness of guidelines, disa­greement with some components, lack of time, resources, motivation, impracti­cality of recommendations, and inertia toward adoption and adherence are some of the factors identified as reasons for poor compliance with the guidelines. Several methods have been attempted as means of improving compliance, including incorporating sepsis screen protocols into triage systems, creating sepsis carts and checklists, making pre-packed sepsis kits available in the emergency room, organizing multi-disciplinary sepsis re­sponse teams, and implementing sepsis stewardship programs [10-11].

The role of clinical audit systems in implementation is well-established and is based on positive reinforcement through feedback. This study was conducted to analyze the role of a clinical audit system in implementing Surviving Sepsis Campaign guidelines in patients with generalized peritonitis.

## Materials and methods

This prospective observational study was conducted in the emergency department (ED) of a tertiary referral hospital over 21 months. All adult patients (> 18 years) presenting to the ED with generalized peritonitis dur­ing the study period were included. Patients with known rheumatic heart disease, congestive cardiac failure, and patients who received treatment prior to reaching the hospital were excluded. The Institutional Review Board approved this study, and all patients gave written informed consent to participate in the study.

**Figure 1:**
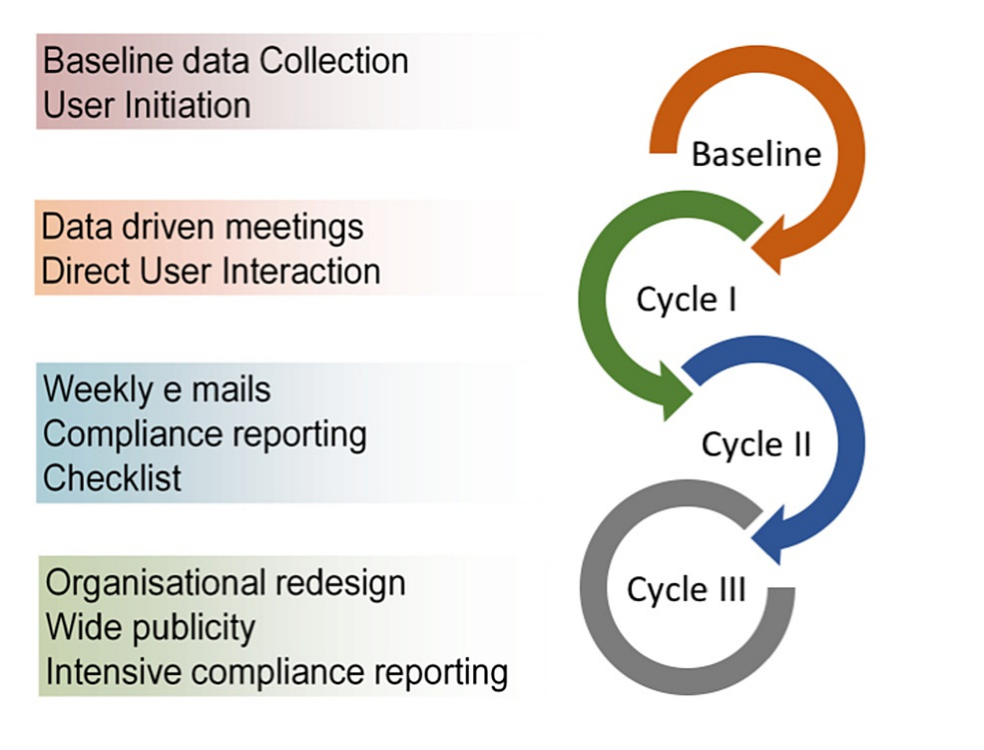
Audit cycle design

The audit was performed in three cycles following a period of baseline data collection. The duration of the cycles was as follows: pre-audit cycle, October to March; audit cycle I, August to December; audit cycle II, January to April; and audit cycle III, May to July (Figure 1).

Auditing for compliance with components of the SSC resuscitation bundle was initiated as soon as the diagnosis of generalized peritonitis was established. The diagnosis of generalized peritonitis was made based on clinical features, radiographic or sonographic pneumoperitoneum, or sonography-guided aspirate of free bowel content, bile, or pus from the peri­toneal cavity.

**Figure 2:**
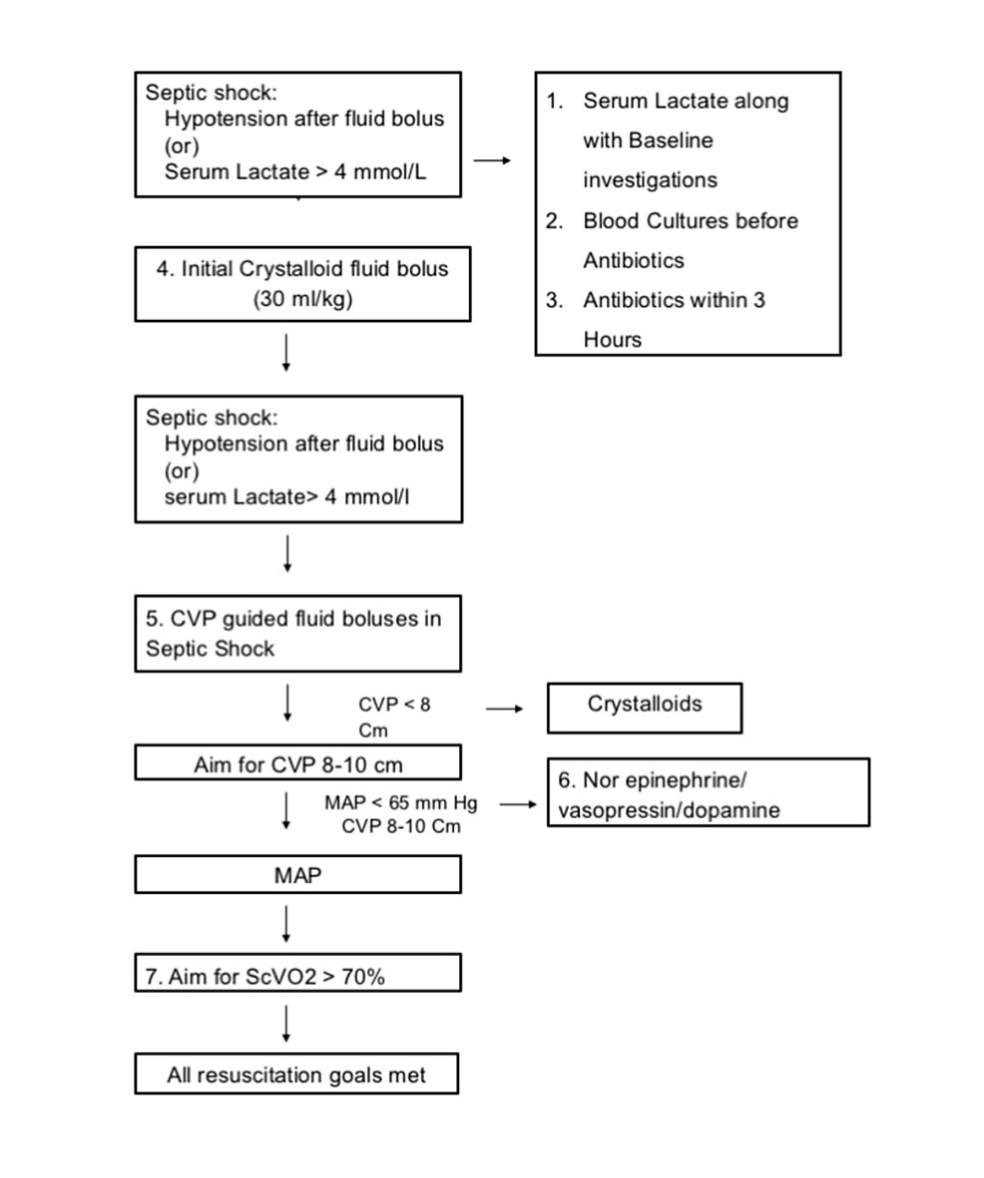
Components of the sepsis bundle

The guidelines followed during the study period are depicted in Figure 2. The first six steps of the guidelines were audited, including measuring serum lactate, blood cultures before antibiotics, antibiotics within three hours, initial fluid bolus, central venous pressure (CVP)-guided fluids when indicated, and inotropes when indicated. The seventh step of central venous oxygen saturation measurement was not included as a requirement owing to logistic reasons.

Compliance data were recorded in four cycles, and each cycle concluded with analysis and feedback. Multi-departmental inputs were sought for process change. The methods for process improvement were implemented as follows:

Pre-audit 

Parameters were recorded to study the baseline compli­ance with the components of the guidelines and identify the areas of poor compliance and the probable causes for the same were presented in this meeting.

Audit I

The SSC coordinator initiated one-to-one communication with individual healthcare professionals regarding audit findings and provided feedback regarding day-to-day compliance.

Audit II

The consultant in charge of the audit sent audit feedback mailed to each consultant and resident. Color-coded checklists were added in the ED to promote compliance with SSC guidelines.

Audit III

Compliance graphs and posters displaying the surviving sepsis resuscitation bundle were put up at all stations. Short group meetings were held with all the team members.

Baseline data collected included patient demographics, date and time of admission, serum lactate, mean arterial pressures (MAP) at presentation, need for inotropes, and the Physiological and Operative Severity Score for the enUmeration of Mortality and Morbidity (POSSUM). Outcome variables were compliance with SSC guidelines, mortality rate, length of ICU stay, and length of hospital stay.

Compliance with each component of the SSC resuscitation bundle and the entire bundle was calculated. A questionnaire-based analysis of reasons for non-compliance was performed.

Statistical analyses

Descriptive statistics were used to compare compliance with each component of the resuscitation bundle in each cycle. Fischer’s exact test and the chi-square test were used to compare categorical variables. One-way analysis of variance (ANOVA) was used to identify a statistically significant difference in the mean values between the two groups (mean ± standard deviation). The Mann-Whitney U test was used for comparing non-parametric data. A two-sided P value of < 0.05 was considered statistically significant. A statistical software package (InStat, Graphpad Software Inc, CA, USA) was used to perform statistical analyses.

## Results

A total of 221 cases of generalized peritonitis were prospectively included in the four cycles of the audit, of which 65 patients were included for baseline data col­lection and 55, 50, and 51 patients were included in audit cycles I, II, and III, respectively. The demographic and baseline characteristics are presented in Table 1.

**Table 1:** Patient characteristics * No statistically significant difference among the groups

	Pre audit (n=65)	Audit (n=55)	Audit II ( n=50)	Audit III ( n=51)
Age	47.7 ± 15.3	48.2 ± 15.0	48.2 ± 15.2	44.1 ± 15.7
Male : Female	5.5 : 1	4.5 : 1	5.6 : 1	5.3 : 1
Physiological score (POSSUM), mean ± SD	26.76 ± 10.13	27.58 ± 9.18	24.81 ± 9.33	Not collected
No. of shocked patients (MAP < 65) at presentation	39 (60%)	29 (52.7%)	21 (42%)	25 (49%)
ICU Stay, mean ± SD	3.2 ± 2.9	3.01 ± 1.45	2.28 ± 1.69	3.1 ± 2.9
Length of hospital stay, mean ± SD	11.9 ± 11.3	11.5 ± 8.6	9.1 ± 5.7	10.9 ± 5.8
Mortality, N(%)	21 (32.3%)	16 (29%)	12 (24%)	11 (20%)

Gastroduodenal perforation was the most common cause of peritonitis in all the audit cycles, fol­lowed by ileal-jejunal, appendicular, colonic, and gall bladder perforations in that order, and the least commonly encountered cause was a ruptured liver abscess.

Only 9.3% of patients presented within 24 hours of acute onset symptoms. About 15.2% of pa­tients presented within 48 hours of the onset of symptoms, 30.2% patients within 72 hours. 45.5% of patients presented after a lag period (time between the onset of acute symptoms to ar­rival at the hospital) of more than 72 hours. A total of 39 (60%), 29 (52.7%), 21 (42%), and 25 (49.01%) patients in pre-audit, audit cycle I, audit cycle II, and audit cycle III, respectively, were in shock (mean arterial pressure (MAP) <65 mm Hg) at presen­tation. The mean POSSUM physiological score at presentation was 26.7 in pre-audit, 27.5 in audit I, and 24.8 in audit II. The score could not be calculated in audit III due to certain logistic reasons. The POSSUM scores at admission in pre-audit, audit I, and audit II were comparable.

Compliance with components of the sepsis bundle

There was 100% compliance with respect to fluid bolus administration, CVP-guided fluid administration, and inotrope use at baseline.

Serum lactate was not performed in pre-audit, audit I, and audit II due to the lack of availability of the lactate measurement kits in the hospital. Serum lactate estimation was made available prior to the start of audit cycle III. Following its availability, serum lactate was performed in 40 patients (78.4%) in audit cycle III.

Blood cultures before antibiotics were obtained only in nine patients (13.8%) in pre-audit, 18 patients (32.72%) in audit cycle I, 30 patients in audit cycle II (60%), and 37 patients (72.5%) in audit cycle III. The improvement was found to be statistically significant in audits I, II, and III when compared to pre-audit values.

Antibiotics were administered within three hours in only 30 patients (46.1%) at baseline, 30 pa­tients in audit cycle I (54.5%), 40 patients in audit cycle II (80%), and 46 patients in audit cycle III (90.1%). The improvement observed in audit II and III was statistically significant when compared to the baseline values (Table 2).

**Table 2:** Compliance with bundle components *p = 0.016, **p < 0.0001, ***p = 0.46, ****p = 0.0002, when compared to pre-audit (baseline) values CVP: central venous pressure

	Pre-audit (n = 65) (%)	Audit cycle I ( n = 55) (%)	Audit cycle II ( n = 50) (%)	Audit cycle III ( n = 51) (%)
Serum lactate measured at admission	0	0	0	40 (78.4)
Fluid bolus administration	65 (100)	55 (100)	50 (100)	51 (100)
Blood cultures before antibiotics	9 (13.6)	18 (32.7)^ *^	30 (60)^**^	37 (72.54)**
Antibiotics within 3 hrs.	30 (46.1)	30 (67.2)***	40 (80)****	46 (90.1)**
CVP-guided fluids	29/29 (100)	16/16 (100)	8/8 (100)	8/8 (100)
Inotropes when indicated	17/17 (100)	13/13 (100)	5/5 (100)	4/4 (100)

The overall bundle compliance increased from 9.2% in pre-audit to 23.6%, 54%, and 64.7% through audit cycles I, II, and III (Table 3).

**Table 3:** Total bundle compliance *Serum lactate was available in the hospital only during audit cycle III

Total number of components performed	Pre-audit ( n = 65) (%)	Audit cycle I ( n = 55) (%)	Audit cycle II ( n = 50) (%)	Audit cycle III ( n = 51) (%)
6/6	0	0	0	33 (64.7)
5/6	6 (9.2)	13 (23.6)	27 (54)	3 (5.8)
4/6	26 (40)	29 (52.7)	16 (32)	11 (21.5)
3/6	33 (50.7)	13 (23.6)	7 (14)	4 (7.8)

User perceptions

Questionnaire-based analysis revealed that 48/50 (96%) participants recognized the importance of performing all the bundle components. Among the responders, the main reasons for non-compliance were lack of time (12%), tedious process (32%), and lack of awareness (12%). The most common reason for non-compliance for blood cultures before antibiotics was the lack of blood culture medium in the emergency department (66.6%).

Outcomes

The overall mortality was found to be 32.3, 29, 24, and 20% in pre-audit, audit cycle I, audit cycle II, and audit cycle III, respectively. The mean hospital stay was 11.95 ± 11.36, 11.5 ± 8.64, 9.16 ± 5.77, and 10.96 ± 5.88 in pre-audit, and audit cycles I, II, and III, respectively. There was no significant difference in the audit cycles in terms of mortality or length of stay.

## Discussion

A major concern in evidence-based medicine is the immense difficulty in translating research from the bench to the bedside irrespective of the level of evidence. This also becomes the main stumbling block in any process improvement program. Surviving Sepsis Guidelines incorporate a bundled care approach in the management of patients with sepsis. Implementing these guidelines is grossly inadequate in our center as it is in many other centers elsewhere. While the actual validity of these guidelines is constantly debated, and the guidelines are regularly revised, this study focused more on the implementation of the guidelines rather than the scientific validity or outcomes related to the guidelines. Accordingly, our observations apply to any process change initiative in healthcare.

This study was organized in the format of a Plan-Do-Study-Act (PDSA) cycle with multiple iterations. Defining the problem ensures that the process improvement team strategizes to achieve maximum success with maximum efficiency. Baseline data generation is an important component in defining the problem and brings out the strengths and weaknesses of the system and its users [12]. The baseline data obtained by us demonstrated our strengths and weaknesses. The data clearly demonstrated that fluid bolus administration, CVP-guided fluid administration, and inotrope use were applied in all cases, resulting in 100% compliance among the components of the sepsis care bundles. The performance of serum lactate at presentation, obtaining blood cultures before antibiotic administration, administering antibiotics within three hours of presentation were infrequently adhered to. The total bundle compliance was a meager 9%.

The three components that had 100% compliance at baseline, namely, fluid bolus administration, CVP-guided fluid administration, and inotrope use, are a part of standard resuscitation for patients presenting with hypovolemic shock. The data suggest that patients with peritonitis were managed as patients in hypovolemic shock, and therefore the components of the guidelines concerning hypovolemic shock were completely adhered to. This data identifies the strengths of the system in having a team that could reliably provide volume resuscitation [13]. The baseline data also demonstrate that specific guidelines for patients in sepsis were not adhered to. The message was to concentrate on training the staff and residents in recognizing the shock in peritonitis as septic rather than hypovolemic shock to the implementing team.

The interventions in audit cycle I aimed at sensitizing residents about the Surviving Sepsis Guidelines. Printed proformas were included at workstations of residents, and one-on-one interaction was conducted to emphasize the need to recognize septic shock and trigger the bundled approach to its management. At the end of this cycle, the total bundle compliance increased from 9% to 23%. A significant improvement was seen both in the number of cases for which blood cultures were sent before antibiotics as well as in the rate of on-time antibiotic administration.

The improvement seen in audit cycle 1 was owing to the action taken by trainees who were already convinced and for the components that did not require much additional effort. In process improvement parlance, this is referred to as reaching for the “low-hanging fruit” [14]. Aiming for easy tasks at the beginning of any process improvement program achieves the twin objectives of boosting process improvement morale as well as winning over easy converts. It might make the task of further process improvement easier by recruiting more campaigners in the form of early converts.

Audit cycle II worked at consolidating the gains achieved over the first cycle, as well as becoming more aggressive in campaigning for compliance with the guidelines. This was done by giving individual user feedback with respect to their compliance. The proformas available at the acute surgery workstations were modified into checklists. Trainees were also encouraged to include the checklists as a part of their patient treatment records [15].

At the end of cycle II, total bundle compliance increased from 23% to 54%. The improvement was seen in the number of cases for which blood cultures were sent before antibiotics, as well as in the rate of on-time antibiotic administration, which almost doubled from that seen in audit cycle I. This cycle also provided feedback in the form of systemic lacunae in the ED, which prevented full compliance with the guidelines.

An aggressive campaign directed by a person of responsibility or power can serve to increase compliance. However, this reaches a plateau limited by the conditions in the environment, as in the availability or limitations of material resources [16]. Unless the process improvement incorporates systemic changes, compliance is unlikely to improve. Cycle II brought out the systemic deficiencies to the fore, including poor inventory management, and an unconducive environment.

Prior to the start of audit cycle III, major changes in the ED in terms of streamlining the admission process, dedicated earmarking area for surgical admission, ownership of ED admission, and enhancement of nursing and physician presence were carried out at the Institutional level. Along with these changes, the process improvement team conducted small group meetings that included residents and nursing staff to reemphasize the need for following the guidelines.

Serum lactate estimation was made available for the first time in the ED during this cycle. Total bundle compliance was 64%, meaning that from a baseline level where less than one-tenth of the users complied, now more than two-thirds of users were complying with the guidelines. Ninety percent (90%) of patients received on-time antibiotics while blood cultures at admission were sent for 72% of patients. Systemic changes not only made it easier for users to comply with proposed guidelines, but they also convince the user regarding the seriousness of the organization in improving the process. In other words, it is easier to ensure user buy-in of process change if the organization also takes concrete measures towards that process change.

Small group meetings at this stage allowed for a two-way discussion between the process improvement team and the users to understand each other’s viewpoints as well as agreeing upon the next course of action towards realizing shared objectives. This phase was associated with shifting the ownership of implementation from the implementation team to the clinical team.

This study did not demonstrate any improvement in the broad outcomes of mortality and length of stay. The possible reasons for this are two-fold. First, this study was not designed to detect the difference in outcomes. Second, the numbers needed to detect a change in outcomes following the implementation of SSC guidelines far exceed the patient numbers in each audit cycle.

The audit was conducted without undue pressure to modify practice, thus reducing the impact of the “Hawthorne effect" [17]. However, this study had several limitations. The hospital was not equipped with point of care lactate estimation, thus limiting the possibility of achieving full compliance initially. Outcome analysis was not performed in a reliable manner. The process improvement team was an internal team and lacked prior expertise in conducting large-scale process improvement programs.

## Conclusions

A clinical audit system is a dependable tool to implement surviving sepsis campaign guidelines. It brings about improvement by educating users, behavioral modification by providing feedback, and enhances process improvement by identifying and correcting systemic deficiencies in the organization. This study reinforces the importance of a multimodal approach in quality improvement and implementation.
